# Towards the indium nitride laser: obtaining infrared stimulated emission from planar monocrystalline InN structures

**DOI:** 10.1038/s41598-018-27911-2

**Published:** 2018-06-21

**Authors:** B. A. Andreev, K. E. Kudryavtsev, A. N. Yablonskiy, D. N. Lobanov, P. A. Bushuykin, L. V. Krasilnikova, E. V. Skorokhodov, P. A. Yunin, A. V. Novikov, V. Yu Davydov, Z. F. Krasilnik

**Affiliations:** 1 Institute for Physics of Microstructures of RAS, 603950 Nizhny Novgorod, Russia; 2 Lobachevsky State University of Nizhny Novgorod, 603950 Nizhny Novgorod, Russia; 3 Ioffe Institute, RAS, 194021 St.-Petersburg, Russia

## Abstract

The observation of a stimulated emission at interband transitions in monocrystalline n-InN layers under optical pumping is reported. The spectral position of the stimulated emission changes over a range of 1.64 to 1.9 μm with variations of free electron concentration in InN layers from 2·10^19^ cm^−3^ to 3·10^17^ cm^−3^. The main necessary conditions for achieving the stimulated emission from epitaxial InN layers are defined. In the best quality samples, a threshold excitation power density is obtained to be as low as 400 W/cm^2^ at *T* = 8 K and the stimulated emission is observed up to 215 K. In this way, the feasibility of InN-based lasers as well as the potentials of crystalline indium nitride as a promising photonic material are demonstrated.

## Introduction

The interest in indium nitride is mainly due to its narrow band gap (about 0.7 eV)^1,2^, which makes InN a promising material for application in a wide class of optoelectronic devices. With interband optical transitions falling into the 1.5–1.9 μm range, it can supplement the much better studied GaN and AlN and significantly expand possible device applications of III-nitride compounds to the whole spectral range, from UV to near-IR^3,4^. Due to a low effective mass and high electron mobility in InN films, the realization of both optoelectronic and logic components seems feasible; the fabrication of lasers and optical amplifiers for the 1.5–1.9 μm spectral range based on crystalline InN or InGaN compounds would open up exciting prospects for application in communication technologies.

InN growth technology has proven to be the most difficult of all III-nitrides. The first problem in InN epitaxy is the lack of lattice-matched substrates, which results in a dislocation density of at least 10^9^ cm^−2^ in the InN layers. The second problem is the thermal decomposition of InN, which occurs at temperatures above 470 °C. Low growth temperature strongly limits the selection of possible sources of active nitrogen. These factors lead to a high concentration of defects, including electrically active ones^5,6^. As a result, the obtained epitaxial InN is usually a degenerate semiconductor material with an electron concentration of more than 3·10^17^ cm^−3^. The above-mentioned technological problems hamper the study of InN properties. Despite this, considerable progress in device applications of epitaxial InN has been demonstrated recently, including the fabrication of infrared photodetectors^7^, thin-film transistors^8^, photovoltaic converters^9^, and a number of terahertz-range devices^10,11^. One of the possible ways to overcome the lattice mismatch problem is the formation of InN-based low-dimensional structures: nanowires, quantum dots, and quantum “pyramids”^12,13,14^. It has been shown that the equilibrium concentration of free carriers in nanowire structures can be reduced to ~10^13^ cm^−3 ^^15^. The development of near-IR light emitters, photovoltaic devices and infrared photodetectors based on low-dimensional InN structures is the subject of intense study.

Further advances towards practical applications of InN in communication technologies require the fabrication of lasers and optical amplifiers for the 1.5–2 μm spectral range, and the key step in this direction would be the achievement of stimulated emission from crystalline InN. However, to date, stimulated emission from InN has only been demonstrated for a very specific sample, with InN nanobelts grown on a Si(100) substrate^16^. The stimulated emission was observed in a wavelength range of 1.6–1.7 μm under optical pumping at low temperature (20 K) with a threshold excitation power density as high as 75 kW/cm^2^. Despite a demonstration of lasing, no further development has followed, in contrast to the intense study of visible-range InGaN nanowire lasers^17,18^. As for planar epitaxial InN structures, to our knowledge, no report on the stimulated emission has been published so far. On the other hand, rather pure n-InN/GaN/AlN/Al_2_O_3_ planar structures with an equilibrium carrier concentration in the InN layer down to 3∙10^17^ cm^−3^ exhibited intense photoluminescence (PL) corresponding to interband transitions in a degenerate direct-gap material^19,20^. The external quantum efficiency of the spontaneous emission as high as 10% at T = 4.2 K was demonstrated, with a moderate temperature quenching up to the room temperature^21^. Light-emitting diodes based on n-InN/p-GaN/Al_2_O_3_ and n-InN/p-NiO/p-Si heterostructures emitting in the range 1.55–1.6 μm have been obtained^22,23^.

In spite of the sophisticated and demanding growth technique, we consider InN a typical direct-band semiconductor and a promising material for the development of near-IR light-emitting devices. We confirm this by the observation of stimulated emission from crystalline epitaxial InN layers in n-InN/GaN/AlN/Al_2_O_3_ planar waveguide structures under optical pumping. Based on the results of the study of a large number of InN structures with different crystal quality, thickness, composition, growth morphology, and carrier concentration, we determine the necessary conditions for achieving stimulated emission in InN structures.

## Results

In order to obtain stimulated emission and to study the conditions for its realization in the planar InN layers, a series of monocrystalline InN samples were grown by plasma-assisted molecular-beam epitaxy (PA MBE) on (0001) sapphire substrates. The details of the growth procedure can be found in the Method section, *supplementary materials* and elsewhere^24^. To expand the range of the parameters of InN structures that affect the implementation and characteristics of stimulated emission, we have also studied the structures grown earlier at Cornell University. The detailed studies of this series of structures have been previously carried out by several scientific groups, including studies of the temperature and power dependencies of the photoluminescence spectra under CW optical pumping^2,21,25^ and time-resolved transmission measurements^26,27^. We should note that to our knowledge, no attempts were made in previous studies to obtain stimulated emission in InN structures similar to those studied in this work, including PL studies under pulsed optical pumping^28,29^. The samples obtained in IPM RAS and at Cornell University are marked in the text by the prefixes “IPM” and “GS”, respectively.

**Figure 1:**
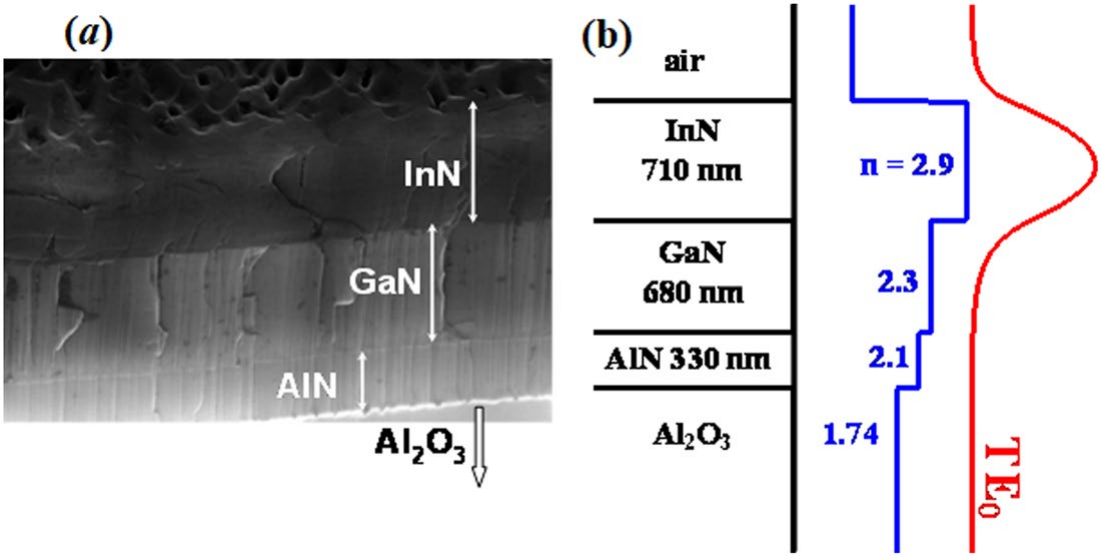
SEM image (**a**) of a typical InN/GaN/AlN/Al_2_O_3_ sample studied (IPM80) and the calculated mode profile (**b**) of the fundamental TE_0_ mode in this planar waveguide.

All samples under study were grown by PA MBE method on c-Al_2_O_3_ substrates. Prior to the deposition of the InN layer a buffer consisting of AlN and GaN layers was deposited onto the sapphire substrate (Fig. 1a). The thickness of GaN/AlN buffer layer was ~1 μm for IPM samples (Fig. 1) and ~0.3 μm for GS samples. Using a GaN/AlN buffer allows partial compensation of the lattice mismatch between InN and Al_2_O_3_. Due to a significant difference in the refractive indices of the active InN layer (n = 2.9), the buffer GaN and AlN layers (n = 2.3 and 2.1, respectively) and c-Al_2_O_3_ substrate (n = 1.74) an asymmetric planar waveguide was formed in the grown structures. The parameters of the InN layer and GaN/AlN buffer in all investigated samples provided strong (>90%) confinement of the fundamental TE_0_ mode in the active InN layer (see Fig. 1b for the calculated mode profile). Optically pumped planar waveguide provides the confinement of the guided light modes in the active layer and eliminates the need to form a resonator for the study of amplification and lasing conditions^30^. The thickness of InN layers was varied in the range 0.5–1.1 μm in the IPM structures, and in the range 1.1–12 μm in the GS structures.

InN structures were investigated by a wide range of the experimental methods in order to reveal the conditions affecting the achievement of stimulated emission in epitaxial InN layers (see section Methods). The performed studies made it possible to determine the morphology of the structures, the roughness of their surfaces, the concentration of the impurity atoms and free charge carriers, the dislocation density, and the presence or absence of the metallic In clusters. In this work, we have analyzed InN layers with the thickness from 0.5 to 12 µm, having both planar and three-dimensional nanopillar structure (see *supplementary materials*), with the dislocation density in the range 10^10^–10^11^ cm^−2^ and the electron concentration from 3∙10^17^ to 3∙10^19^ cm^−3^. According to X-ray diffraction all investigated InN layers were unstrained within the limits of measurement errors. The roughness of the sample surface increased with the thickness of the InN layer with root-mean-square surface roughness values varying from ≤10 nm for the 1 μm thick layers to ≤30 nm, for the layers thicker than 5 μm. The samples under study had an arbitrary shape with the maximal size from 3 to 10 mm. The parameters of some investigated structures are presented in Table 1.

**Table 1:** Parameters of the samples under study and characteristics of the stimulated emission at *T* = 78 K: free carrier concentration (*n*_Hall_), thickness of the active InN layer (*D*_InN_), total density of edge and screw dislocations (*N*_d_), spectral position and width of the stimulated emission line (SE_max_, SE_FWHM_), threshold pump power density (*P*_th_).

Sample	*n*_Hall_, cm^−3^	*D*_InN_, µm	*N*_d_, cm^−2^	SE_max_, meV	SE_FWHM_, meV	*P*_th_, kW/cm^2^
IPM34	8·10^18^	0.53	2.3·10^10^	not observed	—	—
IPM36	7·10^18^	0.65	5.1·10^10^	747	19	60
IPM80	1·10^19^	0.71	3.5·10^10^	747	16	65
IPM81	1.8·10^19^	1.1	4.2·10^10^	755	23	330
GS2054	3·10^17^	5.5	1·10^10^	not observed	—	—
GS2060	3.6·10^17^	12.0	2·10^10^	655	2.2	(5–10)
GS2050	5.7·10^17^	7.0	2.9·10^10^	659	7.8	(5–10)
GS1804	7.3·10^17^	1.7	2·10^10^	658	4	5
GS1792	1·10^18^	1.1	5.9·10^10^	672	6.7	(5–10)
GS2042	1.4·10^18^	1.5	2·10^10^	665	5.6	6

The emission spectra were studied under optical excitation using both a continuous wave (CW) laser (up to 500 mW at the wavelength of 0.8 μm) and an optical parametric oscillator, tunable in the spectral range 0.45 to 2.3 μm (up to 3 mJ in 10 ns pulses with a repetition rate of 10 Hz). The emitted radiation was collected from the surface or from the edge of the sample. In case of pulsed excitation, we tuned the pump wavelength in order to achieve uniform excitation of the whole active layer for samples with rather thick InN layers. Taking into account that the intrinsic absorption coefficient of InN exceeds 10^4^ cm^−1^ for the incident light with the wavelength shorter than 1.7 μm, samples with ~ 1 μm thick InN layers were considered optimal for the implementation of stimulated emission. For further details, see the Methods and *supplementary materials*.

**Figure 2:**
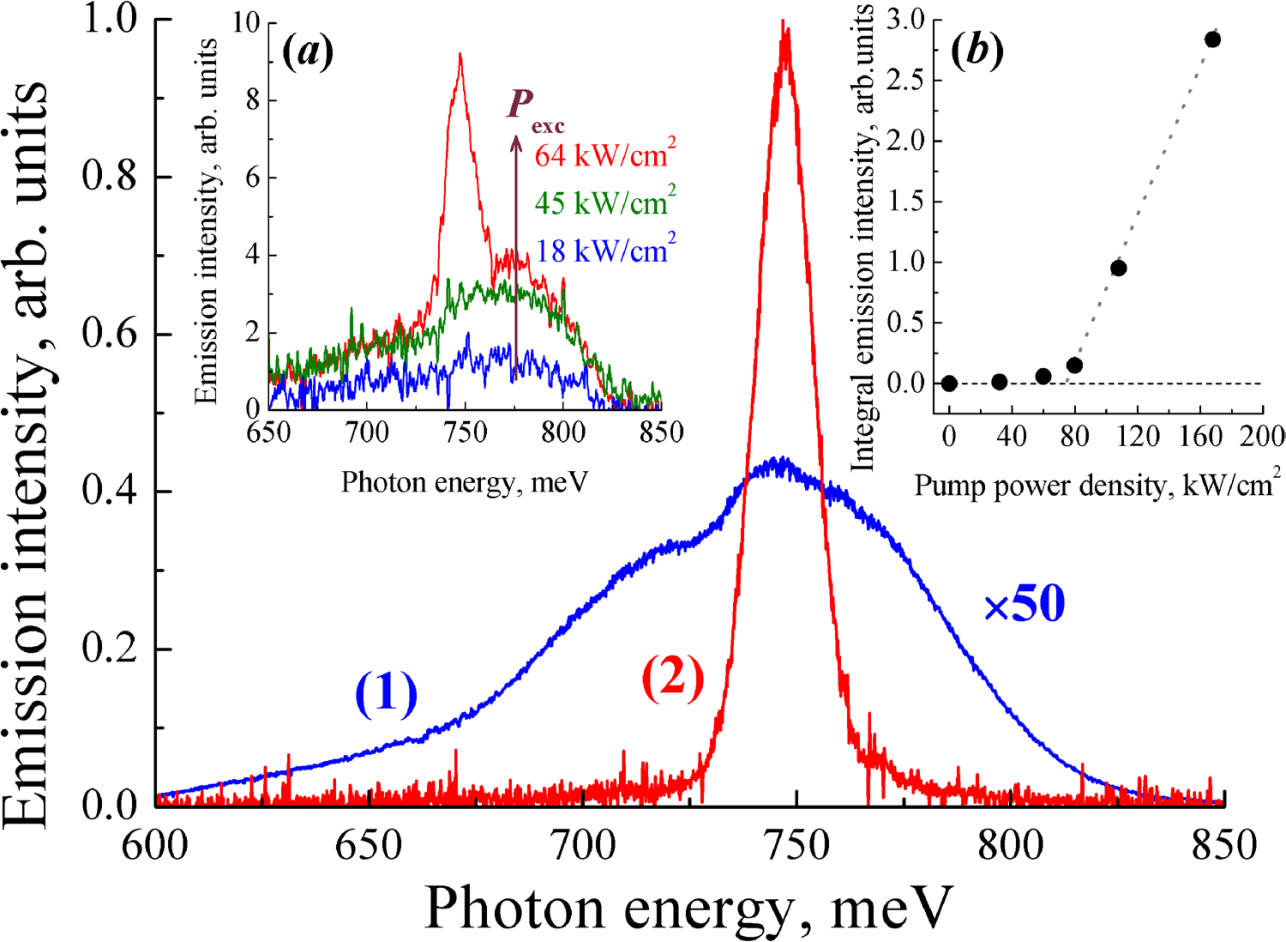
Emission spectra of the sample IPM80 at *T* = 78 K. 1 – CW excitation (λ_ex_ = 800 nm, 1 W/cm^2^); 2 – pulsed excitation (*λ*_ex_ = 1300 nm, 80 kW/cm^2^). Insets: (***a***) transition from spontaneous to stimulated emission near the threshold power density; (***b***) dependence of the integral emission intensity on the pump power density.

The obtained InN/GaN/AlN/Al_2_O_3_ structures demonstrated intense spontaneous PL from the InN layers under CW optical pumping. Figure 2 (curve 1) shows the PL spectrum of the sample IPM80 at *T* = 78 K. The position of the PL peak originated from the band-to-band transitions in InN depended on the concentration of free electrons in the InN layer and demonstrated a blue-shift of the PL maximum for the samples with higher electron concentration in accordance with the change of the Fermi level in a degenerate semiconductor. The low-energy part of the spontaneous emission band, observed in the PL spectra below the band gap energy of bulk InN (<700 meV), was related to the radiative transitions from the conduction band to the localized acceptor states^21^. Under CW excitation, the pump power dependence of the spontaneous PL intensity was sublinear for all samples under study.

Under pulsed excitation, a relatively narrow line (FWHM = 16 meV) arose near the maximum of the spontaneous emission peak at pump power densities above the threshold value (~60 kW/cm^2^ at *T* = 78 K for the sample IPM80, see Fig. 2). The intensity of this line demonstrated a superlinear dependence on the pump power (inset 2 in Fig. 2), which clearly indicated a realization of stimulated emission at the interband transitions in strongly degenerate InN with electron concentration 10^19^ cm^−3^. It should be noted that the use of a planar waveguide without special processing of the facets for the formation of laser cavity mirrors, and the arbitrary shape of the samples resulted in the observation of lasing in a single-pass regime^30^.

**Figure 3:**
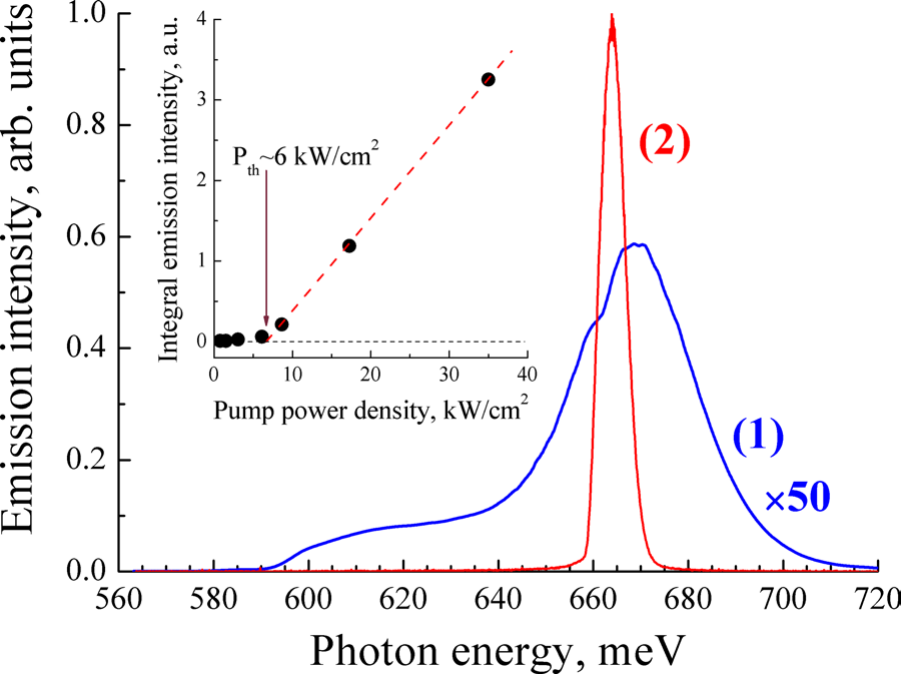
Emission spectra of the sample GS2042 under: 1 – CW excitation (~1 W/cm^2^ at *λ*_ex_ = 800 nm); 2 – pulsed excitation (~9 kW/cm^2^ at *λ*_ex_ = 1300 nm). *T* = 78 K. Inset: integral emission intensity as a function of excitation power density.

**Figure 4:**
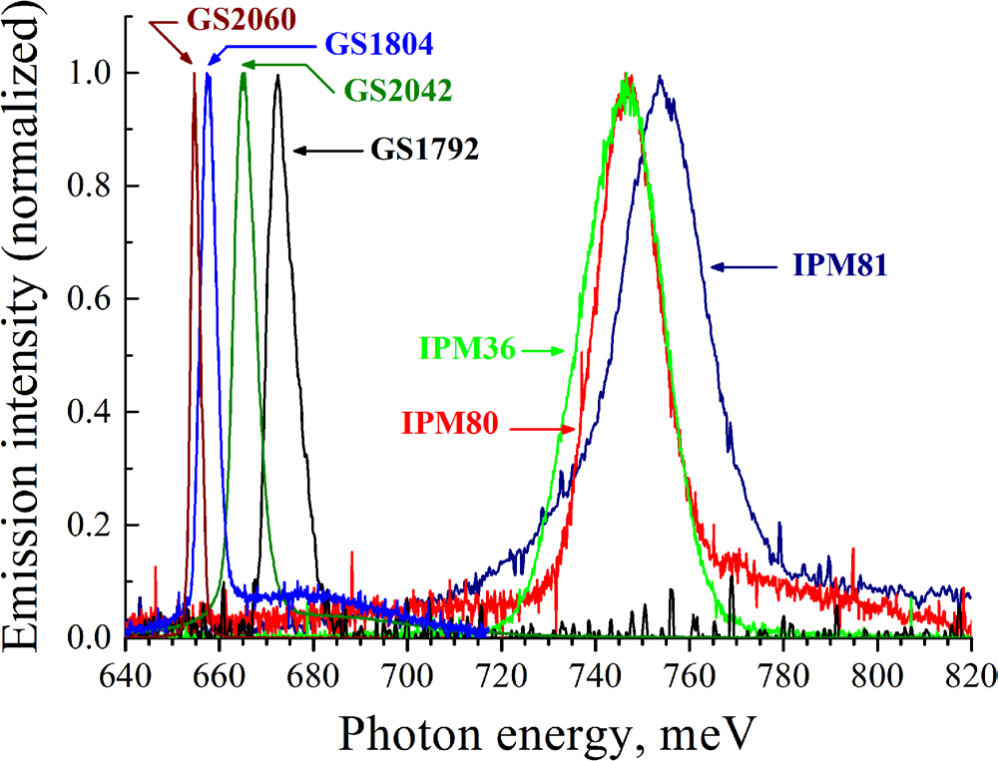
Stimulated emission spectra obtained for different InN samples (see Table 1 for details). All spectra have been measured at *T* = 78 K with excitation power density slightly above the stimulated emission threshold.

**Figure 5:**
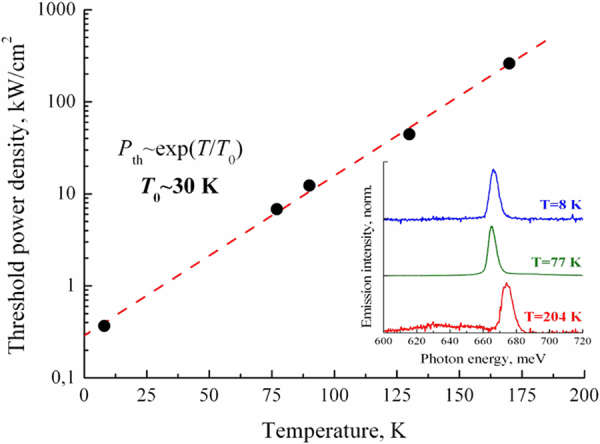
Temperature dependence of the threshold excitation power density for sample GS2042. Inset: stimulated emission spectra at different temperatures. Note the spontaneous emission background visible at *T* = 204 K.

The samples grown in CU had substantially lower electron concentration (see Table 1 and Method section), which led to a strong red shift of the spontaneous emission band and the stimulated emission line (Fig. 3). Figure 4 and Table 1 summarize the results on stimulated emission obtained for all InN samples studied. It can be seen that the change in the concentration of equilibrium electrons in the InN layer from 2∙10^19^ to 3.6∙10^17^ cm^−3^ is accompanied by a shift of the stimulated emission line from 1.64 μm (755 meV) to 1.89 μm (655 meV) and by a substantial narrowing of this line (from 23 meV FWHM down to 2 meV). Besides that, we observed a substantial decrease in the stimulated emission threshold in the structures with lower electron concentration. For the sample GS2042, the estimated threshold at *Т* = 78 K was *P*_th_ ~6 kW/cm^2^ (see inset in Fig. 3), i.e. an order of magnitude lower than in the sample IPM80. This is seemingly caused by a decrease in the non-radiative recombination rate for more pure samples, which is confirmed by a substantial increase in the spontaneous emission intensity in these samples. At *T* = 8 K we estimated a threshold pump power density for the sample GS2042 as low as *P*_th_ = 400 W/cm^2^. For all CU samples with low electron concentration, *P*_th_ was less than 1 kW/cm^2^, which corresponds to an injection current density of <1 kA/cm^2^ in case of electrical pumping. As shown in Fig. 5, at elevated temperatures, the stimulated emission threshold increased exponentially with a characteristic temperature of *T*_0_ ≈ 30 K, which is a limiting factor for high-temperature laser operation. However, for the sample GS2060 with the most pure InN layer, stimulated emission was observed up to 215 K. It should be noted that the threshold of stimulated emission in the obtained planar structures was more than an order of magnitude lower than the previously reported value for the samples with InN nanobelts^16^.

## Discussion

The conditions for optical amplification and stimulated emission can be obtained using the following equation:^30,31^

$$I={I}_{Sp}({e}^{(g-\alpha )l}-1)/g$$

where *I* is the output emission intensity in an edge-emission geometry, *I*_Sp_ is the spontaneous luminescence intensity, *l* is the length of the excited stripe, g is the optical gain coefficient and α is the total losses coefficient including absorption and scattering losses. Consequently, a superlinear dependence of *I*(*l*) for *gl* > 1 is a direct indication of gain and the presence of stimulated emission. Note that the parameters *g* and *l* are determined by the experimental conditions and the intrinsic properties of the active medium, while absorption and scattering losses pertain to deviations from the properties of a homogeneous crystalline layer with a smooth interface. Comparison of the results obtained for a number of planar InN structures made it possible to identify some factors that are essential for the realization of the stimulated emission. The experiment in an edge-emission geometry provided the conditions for obtaining stimulated emission. The power of the pulsed excitation of the active waveguide (in our case this was a planar waveguide, see Fig. 1) was sufficient to create a population inversion and to provide the gain that exceeded the total absorption and scattering losses in a series of InN samples (see Table 1).

At *T* = 78 K, we observed stimulated emission in n-InN/GaN/AlN/Al_2_O_3_ samples with a thickness of the active InN layer of 0.65–12 μm, with a free electron concentration ≤2∙10^19^ cm^−3^ and with a threading dislocation density <6∙10^10^ cm^−2^. As follows from the obtained results, the concentration of free electrons was the most important factor that determines the threshold and temperature dependence of the stimulated emission. In IR light-emitting structures based on degenerate semiconductors, the processes of intraband free carrier absorption are intensified, which leads to an increase in absorption losses, preventing the implementation of lasing. Lower electron concentration resulted in a significant decrease of the threshold power density (see Table 1) and in a higher lasing temperature. The reduction in the electron concentration can be achieved by the growth of thick InN layers^20^, which leads to a decrease in the dislocation density and, as a result, in the concentration of dislocation-related electrically active centers (see samples GS2050, GS2060, GS2054). Besides that, the concentration of free electrons is strongly affected by the amount of residual impurities in the InN layer. Secondary-ion mass spectrometry (SIMS) analysis had shown that the InN layer grown in CU and characterized by lower concentration of equilibrium electrons as compared with IPM samples, also had a lower concentration of residual impurities, primarily oxygen and carbon (*see* Supplementary Fig. S5) Interstitial carbon and substitutional oxygen atoms act as donors and can contribute to n-type conductivity of InN^32,33^.

Despite the revealed strong influence of the concentration of equilibrium electrons on the parameters of stimulated emission, an analysis of all the samples studied showed that a low electron concentration is not a sufficient condition for the realization of stimulated emission in InN layers. The results of studying a large number of structures close in geometry and the concentration of free carriers indicate the contribution of scattering processes to total optical losses. It is well known that in semiconductor crystalline waveguides scattering loss may originate from poor crystallinity, mixed growth phases, incorrect stoichiometry or interface roughness^34^. For instance, stimulated emission was not observed in the sample IPM34 (see Table 1), which had one of the lowest values of dislocation density and electron concentration in the InN layer among the IPM samples. The scanning electron microscopy showed that a three-dimensional nanopillar-like growth of InN layer has been realized in the sample IPM34 (see *supplementary materials*) instead of the formation of a continuous planar InN layer as it was observed for the rest of the samples. In this case, strong light scattering in the active InN layer should lead to an increase of optical losses hindering stimulated emission.

**Figure 6:**
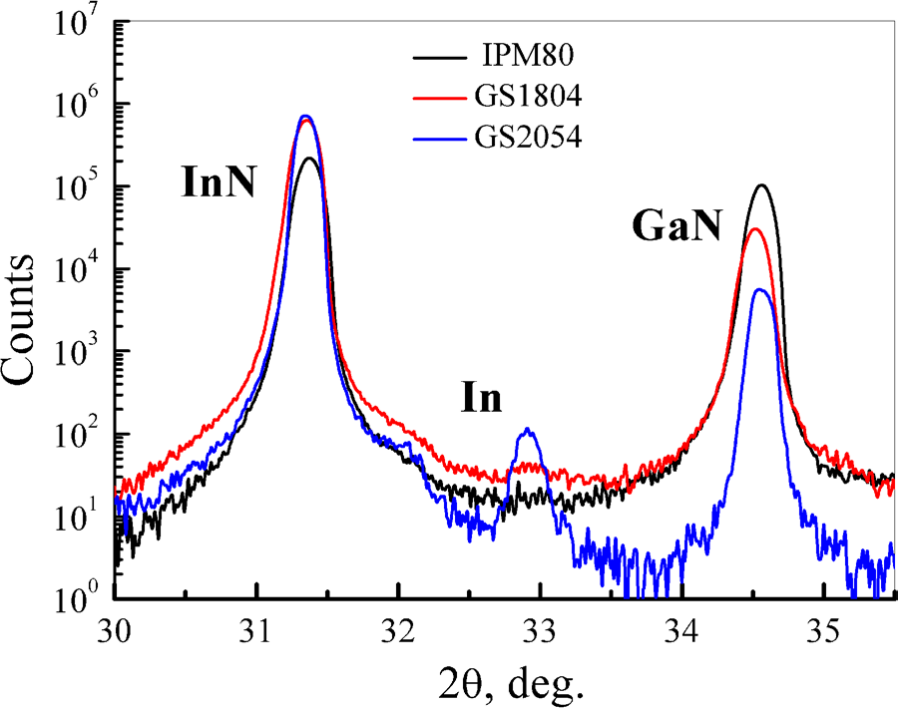
(0002) ω-2θ X-ray diffraction spectra of the samples IPM80, GS1804 and GS2054. The peak from the metallic In phase is denoted by “In”.

Stimulated emission was also not observed in the sample GS2054 despite the lowest dislocation density (10^10^ cm^−2^) and a low electron concentration (3·10^17^ cm^−3^). We have found a factor that can be responsible for the suppression of lasing in this sample. The X-ray diffraction analysis has shown a presence of the metallic In phase. A pronounced peak originating from metallic indium can be seen in the corresponding ω-2θ curve (see Fig. 6). For the samples grown under metal-rich conditions, the formation of metallic In clusters in the InN layer may result in an increase of the radiation scattering. Inclusions of the metallic phase can serve as centers of nonradiative recombination thus also hampering the implementation of lasing.

Thus, along with the low electron concentration and low dislocation density, the basic conditions for the realization of stimulated emission in planar InN structures include the homogeneity of the active InN layer, the absence of the metallic In phase and the low density of the absorption and nonradiative recombination centers. Apart from the optimization of the growth process for InN layers, one can expect further improvement of the lasing characteristics of InN by means of the formation of appropriate waveguide structures and laser cavities.

In summary, stimulated emission at direct band-to-band transitions in crystalline n-InN was obtained under optical excitation. For the best quality samples, the stimulated emission threshold was as low as 400 W/cm^2^ at *T* = 8 K and 6 kW/cm^2^ at *T* = 78 K, and stimulated emission was observed up to 215 K. For epitaxial n-InN films with an equilibrium electron concentration over a range of *n* = 3.6∙10^17^–2∙10^19^ cm^−3^, the emission wavelength varied in the range *λ* = 1.64–1.9 μm. Thus, the feasibility of InN-based laser is demonstrated. In thus way, crystalline indium nitride appears to be a promising photonic material for the realization of a new generation of InN laser structures, active waveguides, microcavities and photonic crystals.

## Methods

### Samples

Samples from the Institute for Physics of Microstructures (IPM RAS) and Cornell University were grown using the PA MBE technique. InN layers were obtained on GaN/AlN buffer layer (~1 μm for IPM samples and ~ 0.3 μm for Cornell University samples) on c-Al_2_O_3_ substrates. According to atomic-force microscopy study of test structures the root-mean-square surface roughness of the GaN/AlN buffers was less than 1 nm. The total dislocation density in GaN buffers evaluated from X-ray diffraction analysis was in a range (1.5–3.5)∙10^10^ cm^−2^.

The growth of InN layers was carried out at different growth conditions which resulted in a difference of their structural, electrical and optical properties. According to X-ray diffraction all investigated InN layers were unstrained within the limits of measurement errors. The InN layers had the thickness varying from 0.5 to 12 µm, both with planar and three-dimensional nanopillar structure (*see supplementary materials*), and with the dislocation density in the range (1–10)·10^10^ cm^−2^ both for IPM and Cornell University samples. However, InN layers obtained in Cornell University demonstrated lower electron concentration varying from 3∙10^17^ to 1.4∙10^18^ cm^−3^, while in IPM samples it varied from 7∙10^18^ to 3∙10^19^ cm^−3^. This difference could be attributed to a much higher concentration of impurities in the IPM samples (more than order of magnitude for O and C impurities) according to the secondary ion mass spectroscopy (SIMS) (*see* Fig. S5 *in supplementary materials*).

### Samples characterization

The surface of the test structures with a GaN/AlN buffer layer and the investigated InN layer were characterized by atomic force microscopy (AFM) using an NTEGRA Prima microscope and by scanning electron microscopy (SEM) using a Carl Zeiss Supra 50VP microscope. The latter was also used to obtain the cross-sectional images of the samples. X-ray diffraction analysis were performed using a Bruker D8 Discover diffractometer. The half-widths of (0004) and (10–12) reflections were analyzed in order to evaluate the density of screw and edge dislocations in InN layers. The half-width of (0004) reflection from investigated InN layers was varied in range 0.14°–0,76° and the half-width of (10–12) reflection in range 0.27°–0.86°. The total dislocation density was calculated using the technique reported by Moram and Vickers^35^. The photoluminescence (PL) spectra were studied using a Nd:YAG-laser with a wavelength of 532 nm, a grating spectrometer Acton 2300i and an OMA-V photodetector based on a linear InGaAs-photodiode array. Measurements of the reflection and transmission spectra were carried out using a Bruker IFS 125 HR Fourier spectrometer. The above mentioned optical spectroscopy methods together with Hall measurements were used to determine the equilibrium electron concentration in InN layers. The secondary ion mass spectroscopy (SIMS) analysis was performed using a TOF.SIMS-5 system with a time-of-flight mass analyzer. For further details, see *supplementary materials*.

### Emission measurements

We used a standard experimental setup for photoluminescence (PL) studies (see Supplementary Fig. S4). The emission spectra were studied under optical excitation using both a continuous wave (CW) laser (up to 500 mW at a wavelength of 0.8 μm) and an optical parametric oscillator, tunable in the spectral range 0.45 to 2.3 μm (up to 3 mJ in 10 ns pulses with a repetition rate of 10 Hz). The pump beam was focused on the surface of the sample using a cylindrical lens into a stripe 200 μm wide and up to 10 mm long. The emitted radiation was collected from the sample surface in the case of CW excitation, or from the edge of the sample while studying emission under pulse exitation, dispersed using a grating spectrometer (Princeton Instruments Acton 2300i), and detected using an InGaAs-based linear diode array detector OMA-V (sensitivity range 0.8–2.1 μm). Depending on the sample under study, a Ge wafer or a low-pass optical filter were used to filter out the scattered pumping radiation. Most measurements were performed at liquid nitrogen (LN) temperature, with the sample placed into LN Dewar vessel; for temperature-dependent studies, we used a helium closed-cycle refrigerator.

### Data availability

Any additional supporting information may be available from the corresponding author upon request.

## Electronic supplementary material

Supplementary Information
