# Supplementary material for: Towards the indium nitride laser: obtaining infrared stimulated emission from planar monocrystalline InN structures
Source: Sci Rep. 2018 Jun 21;8:9454. doi: 10.1038/s41598-018-27911-2 (PMC6013448; doi:10.1038/s41598-018-27911-2)
Supplement: Supplementary file 1 — Supplementary Information [file 41598_2018_27911_MOESM1_ESM.docx]

**Supplementary materials**

**Towards the indium nitride laser: obtaining of infrared stimulated emission
 from planar monocrystalline InN structures**

B.A. Andreev, K.E. Kudryavtsev, A.N. Yablonskiy, D.N. Lobanov, P.A. Bushuykin, L.V. Krasilnikova, E.V. Skorokhodov, A.V. Novikov, V.Yu. Davydov, Z.F. Krasilnik

The growth of InN samples was performed at the Institute for Physics of Microstructures (IPM RAS) by a plasma-assisted molecular-beam epitaxy (PA MBE) technique using an “STE 3N3” MBE machine (from ZAO “NTO”, St. Petersburg, Russia). Sapphire (c-Al_2_O_3_) substrates were used, with a titanium film deposited onto the back surface of the substrate for heating purposes. Prior to the deposition of the InN layer, a buffer consisting of ~0.3 μm thick AlN and ~0.8 μm thick GaN was deposited onto the substrate. For the obtained GaN/AlN buffers, the root-mean-square surface roughness was smaller than 1 nm, and the total dislocation density amounted to (1.5–2.5)×10^10^ cm^–2^, according to X-ray diffraction analysis (see Figure S1). A Bruker D8 Discover diffractometer was used for X-ray diffraction analysis. For samples IPM34 and IPM36, the nitrogen flux during the growth of InN layer was [N] ~ 0.5 μm/h; indium fluxes were set to [In]/[N] ~ 0.5 for sample IPM34 and [In]/[N] ~ 1 for sample IPM36. For the first 5 min of the InN layer growth, the growth temperature was ~ 420°C; after 5 min this was increased to ~ 480 °C. The total time for InN layer deposition amounted to 2 h.

**
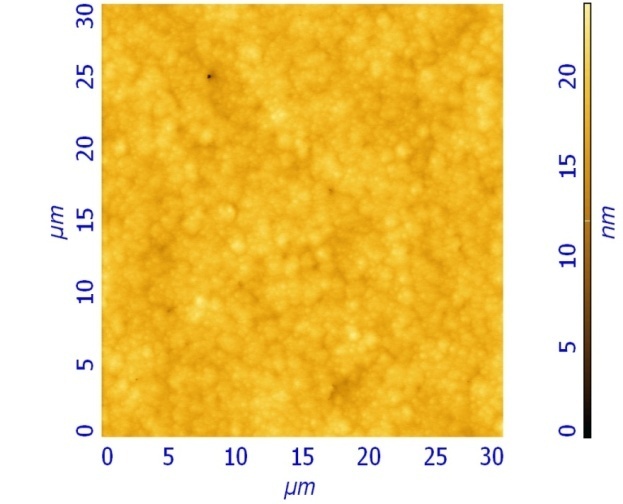
**

**Figure S1.** Left side: typical (0002) ω-2θ X-ray diffraction spectra for IPM samples under investigation; no indication of a metallic indium phase can be seen. Right side: AFM image of the surface of the GaN buffer layer prior to InN growth.

A Carl Zeiss Supra 50VP microscope was used for scanning electron microscopy (SEM) studies. Figure S2 shows the SEM images of InN samples grown with different III-V fluxes. One can see that sample IPM34 exhibits three-dimensional nanopillar-like growth; in this case, strong light scattering in the “active layer” can be expected. A solid InN layer is visible for sample IPM36, and this favors the achievement of stimulated emission.


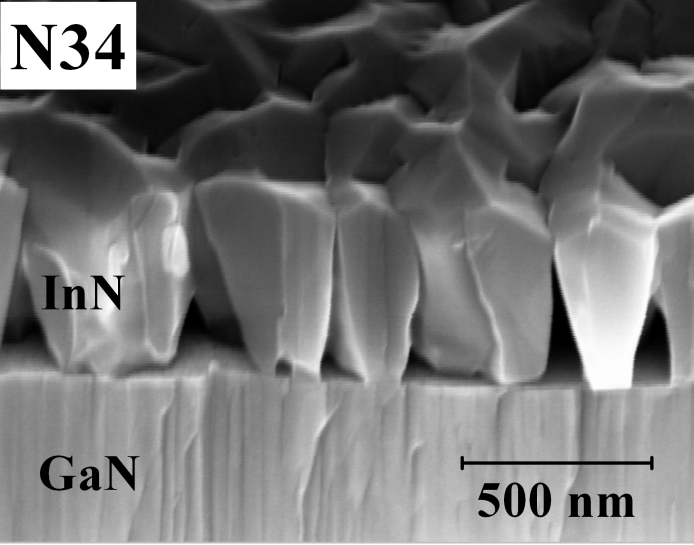

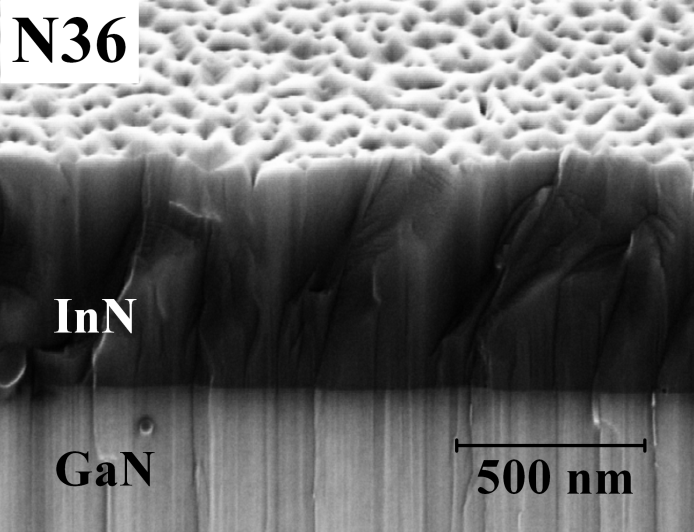


**Figure S2**. SEM images of InN/GaN/AlN/Al_2_O_3_ samples IPM34 (left) and IPM36 (right)

For samples IPM80 and IPM81, a metal migration epitaxy (MME) technique was implemented for the growth of InN layers. The idea of MME growth is to tune the III/V-ratio through the periodical modulation of [In] and [N] fluxes (the corresponding temporal chart is shown in Figure S3). This allows for In-rich growth conditions, which is preferable due to the enhanced surface diffusion of [In] adatoms (resulting in better crystalline quality), while the formation of metallic [In] nanoclusters is suppressed. The growth temperature was 440°C for IPM80 and 450°C for IPM81.The total thickness of InN was ~ 0.8 µm for both samples.

**Figure S3.** Temporal dependence of In and N fluxes during growth of samples IPM80 and IPM81.

**
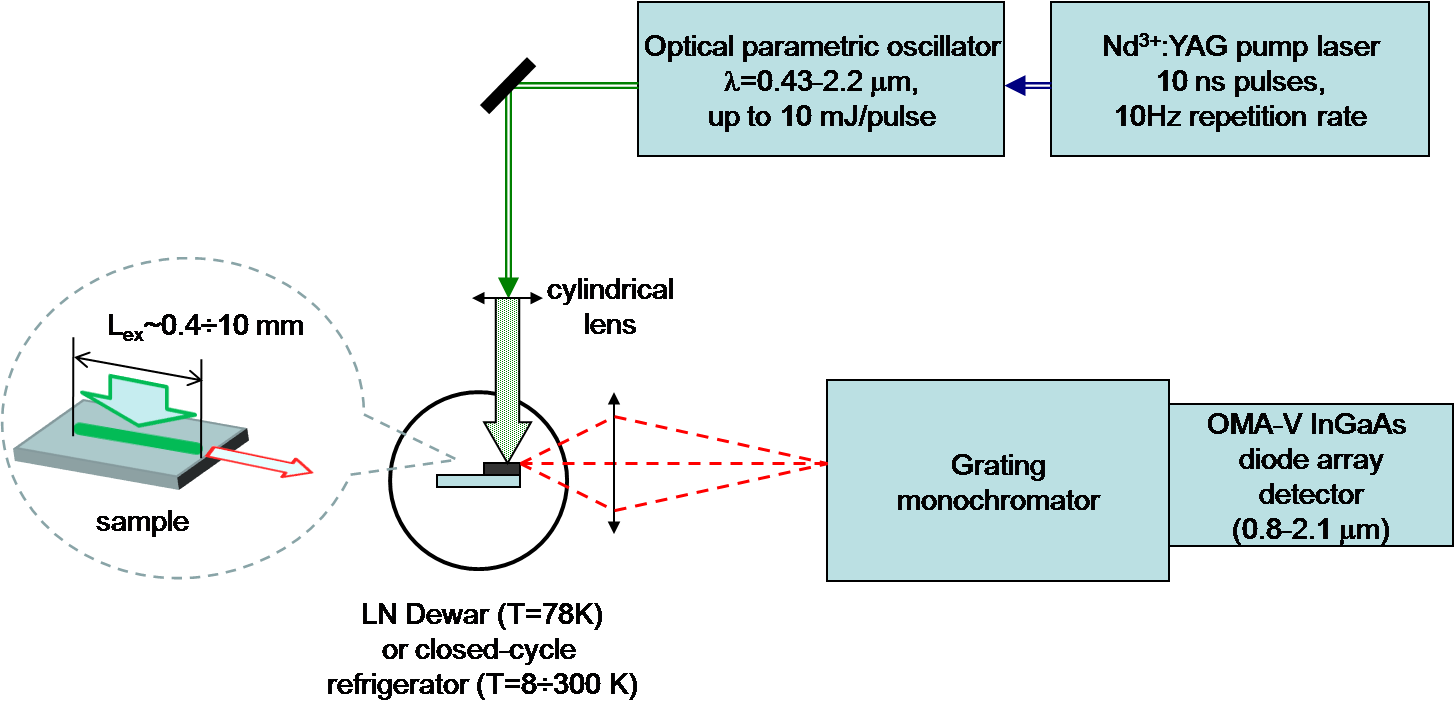
**

**Figure S4.** Experimental setup for photoluminescence studies

**Figure S5.** Oxygen (curves 1 and 3) and carbon (curve 2) atom distribution profiles in InN layers grown in IPM (curves 1 and 2) and CU (curve 3). The level of carbon atoms in analyzed CU structures is less than detection limit of SIMS. The arrows indicate the end of InN layer in the relevant structure.
